# Endoscopic Ultrasound-Directed Transgastric Endoscopic Retrograde Cholangiopancreatography (EDGE): Techniques, Outcomes and Safety Profiles

**DOI:** 10.3390/jcm14165675

**Published:** 2025-08-11

**Authors:** Filippo Antonini, Giacomo Emanuele Maria Rizzo, Giuseppe Vanella, Lorenzo Fuccio, Andrea Lisotti, Michiel Bronswijk, Enrique Pérez-Cuadrado-Robles, Cecilia Binda, Stefano Mazza, Andrea Anderloni, Carlo Fabbri, Ilaria Tarantino

**Affiliations:** 1 Gastroenterology and Interventional Endoscopy Unit, Mazzoni Hospital AST Ascoli Piceno, 63100 Ascoli Piceno, Italy; 2 Gastroenterology and Endoscopy Unit, Istituto Mediterraneo per i Trapianti e Terapie ad Alta Specializzazione, IRCCS-ISMETT, 90127 Palermo, Italy; 3 Pancreatobiliary Endoscopy and EUS Division, San Raffaele Scientific Institute IRCCS, 20132 Milan, Italy; 4 Department of Medical Sciences and Surgery, University of Bologna, 40138 Bologna, Italy; 5 Gastroenterology Unit, IRCCS-Azienda Ospedaliero-Universitaria di Bologna, 40138 Bologna, Italy; 6 Gastroenterology Unit, Hospital of Imola, University of Bologna, 40026 Bologna, Italy; lisotti.andrea@gmail.com; 7 Gastroenterology and Hepatology, Imelda Hospital, 2820 Bonheiden, Belgium; 8 Gastroenterology and Hepatology, KU Leuven University Hospitals Leuven, 3000 Leuven, Belgium; 9 Department of Gastroenterology, Georges-Pompidou European Hospital, 75015 Paris, France; 10 Gastroenterology and Digestive Endoscopy Unit, Forlì-Cesena Hospitals, AUSL Romagna, 47121 Forlì-Cesena, Italy; cecilia.binda@gmail.com (C.B.);; 11 Gastroenterology and Digestive Endoscopy Unit, IRCCS Foundation Policlinico San Matteo, 27100 Pavia, Italy; 12 Department of Internal Medicine and Medical Therapeutics, University of Pavia, 27100 Pavia, Italy

**Keywords:** endoscopy, EDGE, endoscopic ultrasound directed transgastric endoscopic retrograde cholangiopancreatography, ERCP, EUS, endoscopic ultrasound, interventional, LAMS

## Abstract

Patients with Roux-en-Y gastric bypass (RYGB) are a significant challenge for endoscopic retrograde cholangiopancreatography (ERCP) due to the altered anatomy. Endoscopic ultrasound (EUS)-directed transgastric ERCP (EDGE) has emerged as a valuable alternative to standard methods like enteroscopy-assisted (EA-ERCP) and laparoscopy-assisted (LA-ERCP) ERCP. EDGE involves creating a temporary fistula between the gastric pouch and the excluded stomach under EUS guidance, typically using a lumen-apposing metal stent (LAMS). This allows a standard ERCP scope to access the second duodenum and the biliary tree with standard devices. Several studies have investigated the efficacy and safety of this approach, with variations in techniques such as suturing the LAMS to prevent migration. EDGE has demonstrated high technical success rates, and current evidence indicates that it can be performed safely, with acceptable rates of adverse events such as stent migration, bleeding, and perforation, making it the preferred option in referral centers. This comprehensive review aims to provide a concise evaluation of EDGE, its techniques, outcomes, and role in managing biliary and pancreatic disorders in RYGB patients.

## 1. Introduction

Obesity is a significant public health issue, with rising prevalence and a clear association with a variety of medical comorbidities, including cardiovascular diseases, diabetes, and certain cancers [1,2]. Roux-en-Y gastric bypass (RYGB) has become one of the most widely performed bariatric procedures due to its effectiveness in promoting substantial weight loss and improving outcomes of obesity-related comorbidities [3]. However, despite its efficacy in weight management, RYGB increases the risk of developing biliary diseases. Approximately 35% of patients develop gallstones within 18 months post-surgery, significantly raising their likelihood of requiring endoscopic retrograde cholangiopancreatography (ERCP) [4,5]. In addition, the majority of biliary complications consist of chronic or acute cholecystitis, with about 12.2% developing biliary pancreatitis and 5.7% developing choledocholithiasis [6]. Altered anatomy in RYGB precludes conventional ERCP because of the need to transverse the jejunum to access the duodenum and ampulla. Therefore, enteroscopy-assisted ERCP (EA-ERCP) and laparoscopy-assisted ERCP (LA-ERCP) have been developed to address these challenges [7,8,9]. While these methods can achieve biliary drainage [10], they are often associated with extended procedural time, suboptimal technical success, increased morbidity, and do not facilitate multiple reinterventions. EA-ERCP has suboptimal technical success in achieving the ampullary/anastomotic region and performing effective therapeutics, as it also involves endoscopes with no elevator and smaller operative channels than duodenoscopes. LA-ERCP, despite demonstrating higher success rates compared to EA-ERCP, involves a surgical intervention and its associated risks, and requires logistical adaptation of the endoscopy team and equipment with fluoroscopy in the surgical theatre [11]. On the other hand, endoscopic ultrasound (EUS)-directed transgastric ERCP (EDGE) has emerged as a promising alternative in the era of advanced EUS-guided interventions since 2014 [12,13]. By creating a gastrogastric or jejunogastric connection under EUS guidance, this procedure enables access to the excluded stomach and allows for conventional ERCP to be performed without the need for surgical intervention [14]. The EDGE has gained increasing favor due to its high technical success rates, shorter procedure durations, and its ability to be performed entirely within the endoscopy room [15,16]. Nevertheless, widespread adoption of this technique necessitates a comprehensive understanding of its indications, procedural steps, safety considerations, and long-term outcomes. The aim of this narrative review is to explore the principles, technical findings, clinical outcomes, and current evidence of EDGE, highlighting its role in the management of RYGB patients requiring biliary interventions.

## 2. Technical Overview of the EDGE Procedure

A critical element of the EDGE procedure is the use of a specific stent for fistula creation, the lumen-apposing metal stent (LAMS), which ensures the maintenance of a stable and secure connection, thereby providing reliable access to the remnant stomach. However, high expertise in both interventional EUS and ERCP techniques is needed for minimizing adverse events (AEs) [17], which may arise during gastrointestinal anastomoses as well as during subsequent ERCP procedures [18,19]. Notably, the introduction of the “electrocautery-enhanced LAMS” (EC-LAMS) has simplified the EUS-guided procedure by reducing the number of procedural steps, eliminating the need for guidewires and tract dilation, thereby optimizing the process and enhancing procedural efficiency and safety [20]. The EDGE procedure involves three main key steps: first, under EUS guidance, a gastro-gastrostomy or jejuno-gastrostomy is created by deploying a LAMS; second, a conventional ERCP is performed using a duodenoscope through the newly established tract; and finally, the LAMS is removed. Peri-procedural management includes a careful evaluation of the clinical status of the patient and the availability of the instrumentation. Overall, the use of carbon dioxide insufflation is recommended to minimize the risk of gas-related complications [21], while the administration of antibiotics is not routinely recommended but may be considered based on technical difficulties and clinical indications, such as the presence of cholangitis [22]. No specific recommendations are given for the type of sedation, so the procedure is typically performed under general anesthesia or deep sedation, based on internal institutional policies and the anesthesiologist’s preference.

**Figure 1:**
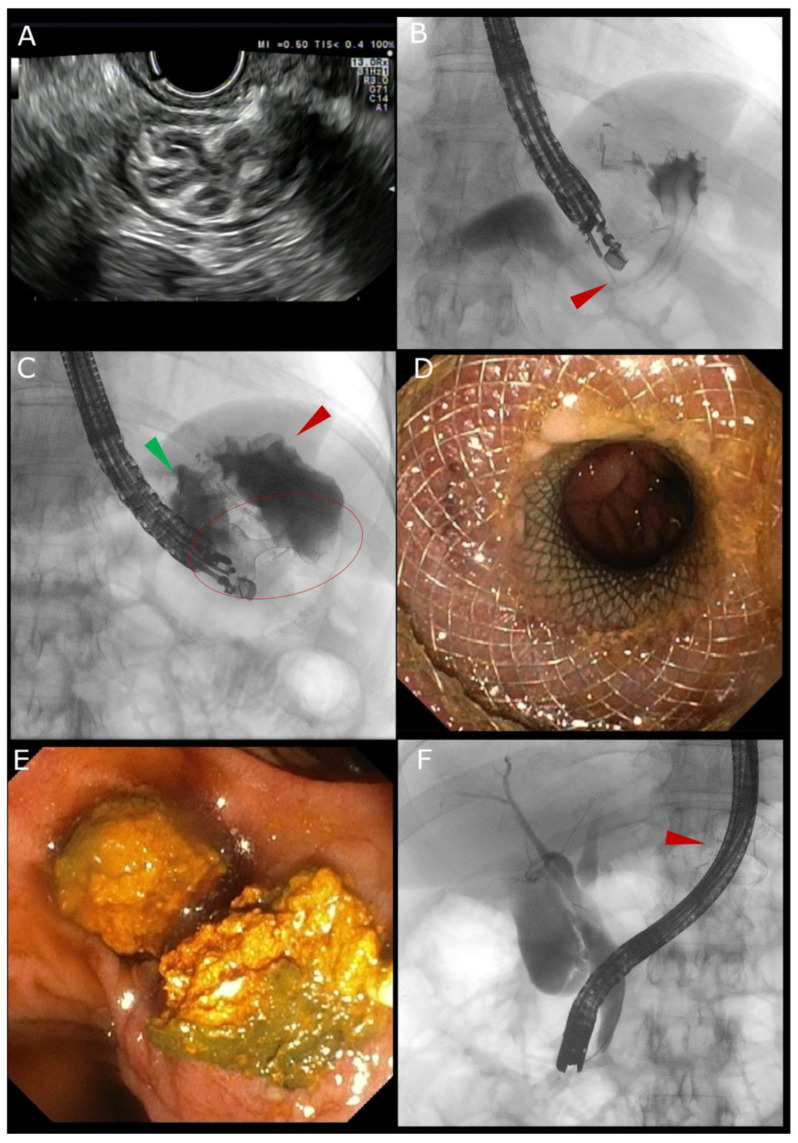
(**A**) Endoscopic ultrasound (EUS) view of the “sand dollar sign” for the identification of the excluded stomach. (**B**) EUS-guided contrast injection through the needle (red arrow) confirmed access to the gastric remnant by radiological view. (**C**) A 20 × 10 mm lumen-apposing metal stent (LAMS, red circle) was then placed between the gastric pouch (green arrow) and the excluded stomach (red arrow). (**D**) Endoscopic view of the excluded stomach through the LAMS. (**E**) Endoscopic view of the biliary stones extracted through conventional ERCP. (**F**) Radiologic view of the conventional ERCP conducted by passing a duodenoscope through the LAMS (red arrow).

A proposed clinical guide to the EDGE procedure is shown in Table 1. Figure 1 illustrates the standard step-by-step approach to EDGE.

### 2.1. EUS-Guided LAMS Placement

A therapeutic linear echoendoscope is advanced into the gastric pouch or proximal Roux limb to visualize the excluded stomach sonographically, the identification of which is crucial, requiring differentiation from adjacent small bowel loops. The EUS-view of the collapsed or flattened antrum is known as the “sand dollar sign”, helping pinpoint the correct target for creating a fistula through the placement of the LAMS. Doppler ultrasound is used to avoid vascular structures, and the distance between the two lumens should be short enough to allow the deployment of the LAMS, approximately 10 mm. Once a safe access window is confirmed, the excluded stomach is first punctured using a fine-needle aspiration (FNA) needle so that saline mixed with contrast is injected under fluoroscopic guidance to verify appropriate needle positioning. If needed, a higher volume of fluid may be instilled to distend the excluded stomach, with the goal of gastric wall separation for accepting the LAMS [23,24]. At the operator’s discretion, a colorant such as indigo carmine or methylene blue may be added to the infusion to aid in confirming correct stent placement after its release, similarly to the technique used in EUS-guided gastroenterostomy [25]. An EC-LAMS is then deployed between the gastric pouch and the remnant stomach, either over a guidewire, if one was previously introduced through the needle used to puncture the remnant stomach, or using the freehand technique, depending on operator preference [26]. However, the site of needle injection and LAMS placement might be different, and, in case of inadequate distention, the patient’s position might be changed. Indeed, to facilitate smooth passage of the duodenoscope and maintain normal anatomical alignment, the LAMS should ideally be placed from the pouch into the proximal gastric body, as extreme positions towards the fundus or antrum have been associated with more difficult ERCP [27]. The LAMS must have a minimum diameter of 15 mm (but 20 mm would be even better) to ensure the safe passage of the duodenoscope, thereby reducing the risk of leakage and stent dislodgement [23,24,28]. The final positioning of the stent is confirmed fluoroscopically with contrast injection.

### 2.2. ERCP via the Created Access

**Figure 2:**
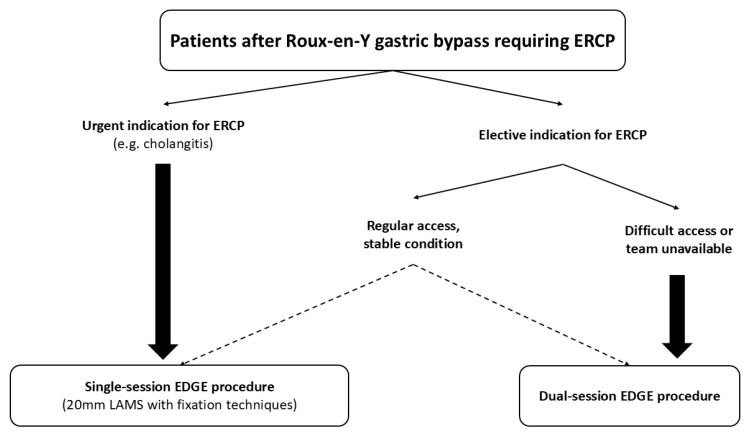
Decision-making algorithm for selecting single vs. dual-session EDGE procedure.

ERCP can be performed either during the initial procedure, called single-session EDGE (SS-EDGE), or in a delayed separate approach (“staged” or “two-stage” or “dual session” EDGE) (Figure 2). Staged EDGE involves a brief maturation period of typically at least one week following LAMS deployment. During this period, the created fistula becomes more stable, reducing the risk of adverse events such as leakage or perforation. After the tract has adequately matured, a therapeutic duodenoscope is introduced through the LAMS into the residual stomach and advanced toward the second duodenum to access the major papilla for ERCP [29,30]. If the lumen of the LAMS is not sufficiently dilated, balloon dilation can be performed, usually up to the maximum size of the stent diameter, to facilitate passage of the duodenoscope. In case the ERCP cannot be deferred (for example, for cholangitis), LAMS fixation, with over-the-scope clips (OTSCs) or suturing devices, can help stabilize the position of the stent and reduce the risk of intraprocedural migration and peritonitis, as discussed in more detail below (Section 4.1) [31,32].

### 2.3. Other Procedures Through the LAMS

In addition to standard ERCP procedures, such as biliary cannulation, sphincterotomy, stone extraction, and stent placement, the EDGE-created fistula also provides access for other advanced interventions, so-called EUS-directed transgastric intervention (“EDGI”), that includes endoscopic biopsy of gastroduodenal luminal abnormalities, EUS-fine needle biopsy (FNB), EUS-guided biliary drainage, and EUS-guided cystogastrostomy [30,31,33,34]. Moreover, access through the LAMS can also facilitate the hemostasis of bleeding lesions located in the excluded stomach or duodenum, areas that are otherwise inaccessible. This includes treatment of bleeding ulcers, angiodysplasias, or anastomotic lesions in post-RYGB patients [35].

### 2.4. LAMS Removal

Following successful ERCP, the LAMS is typically removed after four weeks, whenever additional interventions are not foreseen [36]. Overall, two primary approaches have been proposed for addressing fistulas following LAMS removal. The first involves primary closure, utilizing a variety of techniques such as endoscopic suturing, through-the-scope clips, or OTSCs, with or without previous argon plasma coagulation (APC). These methods aim to achieve immediate and direct sealing of the fistula, even if other unconventional methods have been reported. In fact, some authors adapted devices from different fields (e.g., interventional cardiology) for closing the persistent fistula tract, such as a cardiac septal occluder [37,38].

The second approach involves a wait-and-see approach, sometimes with the application of APC only, to promote secondary intention healing, as the long-term LAMS indwelling seems necessary for maintenance of the fistula. Fistula closure can then be confirmed through endoscopy or upper gastrointestinal series [39].

However, in certain clinical situations, the LAMS may either be left in place or replaced with double pigtail plastic stents [23,40], especially in patients needing long-term access to the remnant stomach or those with malignancies [41,42], where a permanent reversal of RYGB could help prevent excessive weight loss and improve nutritional support.

## 3. Technical and Clinical Success

The EDGE procedure has demonstrated high technical and clinical success rates in patients with RYGB anatomy (Table 2), outperforming other established approaches such as EA-ERCP and LA-ERCP, considering all the outcomes [8]. Runge et al.’s multicenter study [28] reported a technical success rate of 98% on 178 patients with a mean procedure time of 92 min. A high number of studies have been reported so far for evaluating EDGE [16,24,27,28,31,43,44,45,46,47,48,49,50,51,52], and even comparative studies with other approaches are available [25,53,54,55,56,57]. Moreover, one of the alternatives, LA-ERCP, achieves superior therapeutic success when compared to EA-ERCP, with reported rates of 97.9% versus 73.2%, respectively [29,58].

Despite its higher efficacy, LA-ERCP carries a higher risk of adverse events (AEs) compared to EDGE (19.0% vs. 6.5%) and is associated with significantly longer procedural durations (158.4 min vs. 100.5 min) [29]. However, it must be mentioned that LA-ERCP does allow for same-session cholecystectomy in patients with a naïve gallbladder, reducing the procedural burden for this particular patient group. A meta-analysis comparing the effectiveness and safety of three techniques found that EA-ERCP was the least effective, with an overall success rate of 87.3%, duct cannulation 74.7%, and therapeutic success 69.1%. In contrast, EDGE and LA-ERCP demonstrated significantly higher success rates (EDGE: 97.9%, LA-ERCP: 99.1%), along with similarly high cannulation and therapeutic success [60]. A recent network meta-analysis showed that EDGE has a superior technical success compared to EA-ERCP (OR: 4.507, *p* < 0.001), comparable success to LA-ERCP (OR: 0.768, *p* = 0.704), and significantly shorter procedural time than both EA-ERCP (mean difference: −31 min, 95% CI: −40.748 to −21.217, *p* < 0.001) and LA-ERCP (MD: −78.145 min, 95% CI: −104.882 to −51.407, *p* < 0.001), without an increase in AEs [8]. Other meta-analyses have been published, confirming the latter results [7,8,11,60,61,62,63,64] (Table 3).

## 4. Safety

EDGE has demonstrated favorable outcomes in terms of both efficacy and safety, although, like all advanced EUS-guided procedures, it carries a risk of complications. Reported AEs include LAMS migration or dislodgement (15–16%), bleeding (6–7%), perforation (2–4%), and post-ERCP pancreatitis (PEP) (2%) [7,8,43]. Most of these AEs are mild and can be managed conservatively or with endoscopic interventions. Surgical intervention is rarely required. Recent meta-analyses have further assessed the safety profile of EDGE in comparison with LA-ERCP and EA-ERCP [8,64]. Although some studies [60] report lower AE rates with EA-ERCP, these discrepancies are likely due to methodological differences, including variations in patient selection, outcome definitions, the inclusion of retrospective non-comparative studies, and inconsistent reporting of outcomes by surgery type across studies. Despite these variations, only modest differences in overall complication rates have been observed among the three approaches: EDGE (13–21%), LA-ERCP (16–17%), and EA-ERCP (8–10%) [7,64].

### 4.1. Stent Migration/Dislodgement

Stent migration or dislodgement is a significant concern during EDGE, particularly in single-stage procedures. This risk is influenced by LAMS diameter, fixation technique, and patient anatomy. Keane et al. [45], using 20-mm LAMS, which was then sutured to the gastric wall, reported no stent migration. However, other studies have reported varying rates of stent dislodgement. For example, Tyberg et al. [16] reported stent dislodgement in 18.7% of patients during the index procedure, with successful repositioning or bridging in all cases. In a study by Mangiavillano et al. [31], two cases of late stent migration led to gastro-colonic fistulas. However, recent advancements in proximal flange fixation techniques have improved the stability of the stent, which may make SS-EDGE safer by reducing the risk of stent migration [59]. Usually, a 20-mm LAMS is used, which is then dilated to ensure the scope passage. Several methods have been reported to reduce dislodgement of the LAMS, such as mineral oil lubrication of the duodenoscope shaft for reducing friction and preventing migration [57], conventional endoscopic suturing systems, OTSCs [65], and through-the-scope suturing systems [32]. Recently, a dedicated fixation device (Stentfix over-the-scope clip [OTSC], Ovesco Endoscopy, Tübingen, Germany) has been released, which functions as an OTSC but is specifically designed for the fixation of stents, such as LAMS or fully covered self-expandable metal stents. In the multicenter study on the feasibility of this device, no LAMS migration occurred, and technical success was achieved in 95.0% [44]. However, the selection of the optimal stent fixation technique depends largely on the available technical expertise and the resources at hand [59].

### 4.2. Fistula Persistence

Fistula persistence is a potential issue after LAMS removal following EDGE, with ongoing debate about optimal management. However, the clinical relevance of this event is still debated, as weight regain has been rarely described (see below). While spontaneous fistula closure is common, some patients need intervention. Keane et al. [45] reported that a persistent fistula was diagnosed in 25% of patients who underwent objective testing. Factors contributing to persistence include LAMS size, stent duration, allowing the fistula to close by secondary intent, patient healing, and underlying conditions, whereas the location of the fistula and the timing of the ERCP session do not seem to have a significant impact [66]. The management of persistent fistula is similar to that of fistula closure following LAMS removal, and may include APC, endoscopic suturing, and clip deployment [46]. However, there is no consensus on the best approach to prevent fistula persistence. A study analyzing predictive factor for fistula persistence showed that longer LAMS dwell time (mean 127 vs. 48 days, *p* = 0.02) was significantly associated with a higher risk of persistent fistulas in EDGE patients, increasing by 9.5% for every additional seven days in situ, while primary fistula closure and APC treatment were not found to be protective against fistula formation [67]. Moreover, 19 patients among the 25 patients with persistent fistulas underwent endoscopic closure attempts. The techniques used were as follows: OTSC combined with APC in seven patients, endoscopic suturing with APC in seven patients, through-the-scope clips in two patients, endoscopic tacking with APC in one patient, OTSC combined with endoscopic suturing and APC in one patient, and APC alone in one patient. Follow-up assessments showed successful fistula resolution in 14 of these 19 patients (73.7%), all achieved after a single attempt, while the residual 5 underwent multiple endoscopic sessions or were lost at follow-up [67].

Mucosal ulceration at the LAMS site could be an underrecognized but clinically relevant adverse event [28]. It is most likely due to acid exposure and mechanical trauma from the stent. Prophylactic and post-procedural proton pump inhibitors (PPI) therapy could be suggested to prevent this adverse event. [68] In rare, refractory cases, particularly when ulceration persists despite optimal acid suppression, the use of misoprostol may be considered. Its benefit in treating post-bypass anastomotic ulcers has been described and may extend to EDGE-related mucosal injuries [69].

### 4.3. Weight Regain

Weight regain is a potential long-term concern post-EDGE, especially after RYGB. However, current data suggests EDGE does not cause significant weight regain in most patients [57,70]. Tyberg et al. [16] reported a mean weight change of −2.85 kg from LAMS insertion to removal. In Keane et al.’s study [45], patients also had a tendency to lose weight (mean weight loss, 3.1 kg). Another study from Kochhar et al. [56] showed that the mean weight change was negative 6.6 lbs at 28 weeks after the EDGE procedure. Conversely, a weight gain in 63 patients (36.6%) during the time the LAMS was in place was reported in another study, with an average weight gain of 12 lbs, with 38 patients gaining < 5 lbs [43], suggesting a trend toward possible weight gain in patients that did not have their fistula closed at the time of LAMS removal. This variability highlights the need for consistent weight monitoring and data collection across different centers to understand the true risk of persistent fistula leading to weight change after the procedure, and for identifying predictive factors.

## 5. Training Implications for EDGE

Training for the EDGE technique presents unique challenges due to the complex nature of the procedure, which demands proficiency in EUS, ERCP, fluoroscopic guidance, and the deployment of LAMS. Data from a single-operator prospective study involving 19 patients suggests that while technical success can be consistently achieved early on, procedural efficiency improves significantly with experience [48]. In this series, a median procedure time of 54.5 min was reported, with cumulative sum analysis indicating a noticeable gain in efficiency after nine procedures. Furthermore, nonlinear regression analysis demonstrated that procedural times continued to decrease, suggesting a plateau or “mastery” level may be reached after approximately 25 to 35 cases [48]. Although formal training guidelines for EDGE are lacking, these findings underscore the importance of a structured, case-volume-based approach to training. Based on expert consensus and parallels drawn from other interventional EUS procedures, it is recommended that operators have prior experience with at least 50 therapeutic EUS cases, including EUS-guided gastrointestinal anastomoses, and demonstrate competency in complex ERCP (ASGE grade 3–4) before attempting EDGE [71,72,73]. The integration of simulation models and supervised training programs may help accelerate the learning curve and enhance safety during the early adoption phase, though EDGE-specific data on these modalities remains limited [74].

## 6. Economic Considerations

From an economic standpoint, while EDGE may involve higher initial costs due to specialized equipment and technical demands, emerging data suggest it may offer long-term savings by reducing the need for more invasive alternatives such as LA-ERCP or EA-ERCP. A cost-effectiveness analysis found EDGE to be the least expensive and most beneficial option in terms of quality-adjusted life years (QALYs), outperforming both EA-ERCP and LA-ERCP [75]. Moreover, comparative observational data demonstrate substantially lower total procedural and hospitalization costs with EDGE, primarily due to shorter operative times, reduced hospital stays, and minimal need for multidisciplinary coordination compared to LA-ERCP and EA-ERCP [60,62]. Indeed, EDGE’s significantly shorter procedural duration contributes directly to reduced operating room utilization and resource consumption [8,62]. Furthermore, EDGE is associated with shorter hospital stays, typically by over one day, compared to LA-ERCP, further amplifying its cost-saving potential through lower postoperative resource use [11]. Although most available data derive from dual-session EDGE procedures performed in endoscopy units, it is plausible that single-session approaches may yield even greater economic benefits, though formal data are not yet available.

## 7. Conclusions

EDGE in RYGB patients continues to demonstrate high effectiveness and is evolving as a promising method for accessing the biliary tree without the need for surgical revision. Its high technical success rates are associated with high clinical success and a low incidence of severe adverse events. However, it should be noted that to date, literature retrospectively describes the outcomes of a relatively low number of large-volume centers, without any direct head-to-head comparison. Standardization of the technique and awareness of risk factors for AEs seem to have improved procedure outcomes.

To reduce the risk of LAMS migration, new dedicated devices such as the OTSC, as well as different fixation techniques (suturing systems), are increasingly being utilized. However, EDGE requires advanced expertise in both interventional EUS and ERCP techniques, making it the preferred option in specialized referral centers. Further research should focus on refining procedural techniques, evaluating long-term outcomes, and tailoring interventions to individual patient anatomy and clinical context.

## Figures and Tables

**Table 1:** Stepwise guide to performing the EDGE procedure.

Phase	Aspect	Recommendation
**Pre-procedure**	Imaging	Contrast-enhanced CT and/or MRCP to identify remnant stomach anatomy and landmarks
	Antibiotic prophylaxis	Not routinely indicated; advised if cholangitis or complex drainage expected
	Diet	Fast for 8 h pre-procedure
**Intra-procedure**	LAMS selection	Preferably 20 mm LAMS, especially in case of single-session procedure
	LAMS fixation	Consider fixation using OTSC or suturing, especially in case of single-session procedure
**Post-procedure**	PPI	Consider treatment until fistula closure
	Diet	Liquid diet for 24 h, followed by gradual refeeding based on clinical tolerance
	Fistula closure	Remove LAMS at 4 weeks; close fistula if needed via clips or suturing; consider use of APC on the fistula edges

EDGE = EUS-directed transgastric ERCP; CT = Computed Tomography; MRCP = Magnetic Resonance Cholangiopancreatography; PPI = Proton Pump Inhibitor; LAMS = Lumen-Apposing Metal Stent; OTSC = Over-The-Scope Clip; APC = Argon Plasma Coagulation.

**Table 2:** A summary of the studies evaluating outcomes and characteristics of endoscopic ultrasound (EUS)-directed transgastric ERCP (Endoscopic Retrograde Cholangiopancreatography).

First Author	Year	Study Design	Total N/EDGE	Technical Success, n (%)	Clinical Success, n (%)	AEs, n (%)	Severe AEs, n (%)	Comparison with Other Approaches	Sessions
**Bronswijk** [59]	2025	Retrospective	20/20	19 (95)	NA	2 (10)	0	No	Single session (95%); Dual session (5%)
**Rizzo** [40]	2025	Retrospective	216/9	9 (100)	8 (88.9)	1 (11.1)	0	No	Single session (66%); Dual session (34%)
**Mangiavillano** [31]	2024	Retrospective	270/79	77 (97.5)	77 (100)	4 (5.1)	NA	No	NA
**Ghazi** [53]	2024	Retrospective	132/25	22 (89.5)	NA	4 (15.8)	NA	Yes (LA-ERCP, EA-ERCP)	Single session (46.2%) Dual session (53.8%)
**Pérez-Cuadrado-Robles** [27]	2023	Retrospective	33/31	30 (96.8)	30 (100)	1 (3.2)	1 (3.2)	No	Dual session (100%)
**Kedia** [43]	2023	Retrospective	172/172	171 (99.4)	163 (95.3)	48 (27.9)	1 (0.6)	No	Single session (44%) Dual session (56%)
**Keane** [45]	2023	Retrospective	37/37	37 (100)	37 (100)	4 (10.8)	2 (5.4)	No	Single session (100%)
**Chhabra** [46]	2022	Retrospective	14/14	13 (92.8)	13 (100)	3 (21.4)	NA	No	Single session (67%) Dual session (43%)
**Bahdi** [24]	2022	Retrospective	32/32	31 (96.9)	27 (87.1)	11 (34.4)	3 (9.4)	No	Single session (53.1%) Dual session (46.9%)
**Runge** [28]	2021	Retrospective	178/178	175 (98.3)	172 (98.3)	28 (15.7)	4 (2.2)	No	Single session (49%) Dual session (51%)
**Khara** [25]	2021	Retrospective	76/17	17 (100)	17 (100)	1(6)	0 (0)	Yes (LA-ERCP)	Single session (51%) Dual session (49%)
**Wang** [54]	2021	Retrospective	130/18	18 (100)	NA	1	NA	Yes (LA-ERCP, EA-ERCP)	NA
**Barclay** [47]	2022	Retrospective	7/7	7 (100)	7 (100)	0	0	No	Dual session (100%)
**Tyberg** [48]	2020	Retrospective	19/19	19 (100)	18 (94.7)	4 (21.1)	NA	No	Single session (26%) Dual session (74%)
**Kröll** [55]	2020	Retrospective	19/2	2 (100)	NA	0	0	Yes (LA-ERCP, HGS, EDGE)	Dual session (100%)
**Kochhar** [56]	2020	Retrospective	56/26	26 (100)	NA	3 (11.5)	NA	Yes (LA-ERCP, EA-ERCP)	Single session (50%) Dual session (50%)
**de Benito Sanz** [49]	2020	Retrospective	14/14	14 (100)	13 (92.9)	5 (35.7)	NA	No	NA
**James** [50]	2019	Retrospective	19/19	19 (100)	19 (100)	6 (31.6)	0	No	Single session (21%) Dual session (79%)
**Wang** [39]	2019	Prospective	10/10	10 (100)	10 (100)	2 (20)	0	No	Single session (78%) Dual session (22%)
**Kedia** [57]	2019	Retrospective	72/29	28 (96.5)	28 (100)	7 (24.1)	NA	Yes (LA-ERCP)	Dual session (100%)
**Tyberg** [16]	2017	Prospective	16/16	16 (100)	10/11 * (90.9)	4 (25)	1 (6.3)	No	Single session (40%) Dual session (60%)
**Ngamruengphong** [51]	2017	Retrospective	13/13	13 (100)	13 (100)	0	0	No	Single session (15.4%) Dual session (84.6%)
**Attam** [52]	2015	Retrospective	10/10	9 (90)	9 (100)	0	0	No	Single session (100%)

EDGE = EUS-directed transgastric ERCP; EUS = endoscopic ultrasound; ERCP = Endoscopic Retrograde Cholangiopancreatography; AEs = Adverse events; NA = Not available. * only 11 patients out of 16 performed ERCP after EUS-guided gastrointestinal anastomosis creation

**Table 3:** Summary of the meta-analyses available on EDGE (endoscopic ultrasound [EUS]-directed transgastric ERCP [Endoscopic Retrograde Cholangiopancreatography]).

First Author	Year	Pooled Technical Success, %	Pooled Clinical Success, %	Pooled AEs Rates, %	Comparison with Other Approaches
**Gellert** [61]	2025	96	93	20	Yes, vs. LA-ERCP, EA-ERCP
Gangwani * [8]	2024	94.8	NA	14.9	Yes, vs. LA-ERCP, EA-ERCP
**Gangwani** [62]	2024	NA	NA	NA	Yes, vs. LA-ERCP
**Su** [63]	2023	98	94	14	No
**Deliwala** [64]	2023	96	91	17	Yes, vs. LA-ERCP, EA-ERCP
**Gkolfakis** [60]	2022	97.9	97.9	13.1	Yes, vs. LA-ERCP, EA-ERCP
**de Oliveira** [11]	2022	97.8	NA	13	Yes, vs. LA-ERCP
**Dhindsa** [7]	2020	95.5	95.9	21.9	Yes, vs. LA-ERCP and EA-ERCP

* Network meta-analysis. AEs = Adverse events; EDGE = EUS-directed transgastric ERCP; EUS = endoscopic ultrasound; ERCP = Endoscopic Retrograde Cholangiopancreatography.

## Data Availability

Not applicable.
